# A microcosm approach highlights the response of soil mineral weathering bacterial communities to an increase of K and Mg availability

**DOI:** 10.1038/s41598-019-50730-y

**Published:** 2019-10-07

**Authors:** O. Nicolitch, M. Feucherolles, J.-L. Churin, L. Fauchery, M.-P. Turpault, S. Uroz

**Affiliations:** 1 INRA, Université de Lorraine, UMR 1136 “Interactions Arbres Microorganismes”, Centre INRA de Nancy, 54280 Champenoux, France; 2 INRA UR 1138 “Biogéochimie des Ecosystèmes Forestiers”, Centre INRA de Nancy, 54280 Champenoux, France

**Keywords:** Microbial ecology, Element cycles

## Abstract

The access and recycling of the base cations are essential processes for the long-lasting functioning of forest ecosystems. While the role of soil bacterial communities has been demonstrated in mineral weathering and tree nutrition, our understanding of the link between the availability of base cations and the functioning of these communities remains limited. To fill this gap, we developed a microcosm approach to investigate how an increase in key base cations (potassium or magnesium) impacted the taxonomic and functional structures of the bacterial communities. During a 2-month period after fertilization with available potassium or magnesium, soil properties, global functions (metabolic potentials and respiration) as well as mineral weathering bioassays and 16S rRNA amplicon pyrosequencing were monitored. Our analyses showed no or small variations in the taxonomic structure, total densities and global functions between the treatments. In contrast, a decrease in the frequency and effectiveness of mineral weathering bacteria was observed in the fertilized treatments. Notably, quantitative PCR targeting specific genera known for their mineral weathering ability (i.e., *Burkholderia* and *Collimonas*) confirmed this decrease. These new results suggest that K and Mg cation availability drives the distribution of the mineral weathering bacterial communities in forest soil.

## Introduction

Understanding how microorganisms adapt to variations in resource availability is a central question in ecology and evolutionary biology. For plants, different behaviours related to the competition for resources have been described, such as competitors, which adapt to rapidly consume available resources, stress tolerators, which persist in nutrient-poor environments, and ruderals, which adapt to disturbances^1^. These different competitive strategies as well as the related theories have been applied to the analysis of environmental microbial communities^2–4^. However, most of the studies dealing with this question have focused on organic nutrients (C, N, S) or iron. The important role of the composition, quality and recalcitrance of organic carbon compounds in the regulation of the composition of microbial communities has been demonstrated in several aquatic and terrestrial environments^5–7^. Similarly, the form of nitrogen (i.e., ammonium, nitrate, nitrite, amino acids) is known to strongly impact the composition and functioning of microbial communities^8,9^. As an example, N application is known to modify the abundance of nitrifiers and denitrifiers^10^ or methanotrophs^11^. For most of these substrates, the presence and increasing concentrations of compounds directly select microorganisms capable of consuming them or having a better affinity. In contrast, the absence or decreasing concentrations of certain substrates, such as iron, are known to allow the selection of competitive taxa capable of mobilizing those nutrients through the production of siderophores and the regulation of their production^11–13^. Comparatively, the relative impact of base cations on the soil bacterial communities at both the taxonomic and functional levels has been poorly investigated.

Base cations correspond to inorganic compounds, also termed mineral nutrients or inorganic nutrients, such as potassium (K), magnesium (Mg) or calcium (Ca), which act as nutrients, co-factors, or structural components of the cell for plants and the living biosphere^14–16^. These inorganic nutrients are particularly important for plant nutrition, and their depletion can cause plant nutrient deficiencies^14,15^. In both forest and agricultural environments, the concentrations of available K or Mg are low compared to other nutrients (*e*.*g*., C, N) but for different reasons^17^. In agricultural soils, those low concentrations are due to the intensive removal of the crop biomass, but the pool of base cations is usually restored by regular NPK or manure fertilizations. In temperate forest ecosystems, the low concentrations of base cations are due to the nutrient-poor soils in which forests are usually developed, the absence of amendments, and the slow replenishment of the soil fertility^18^. In addition, this phenomenon is accentuated in harvested forests, where trees and the cations accumulated in their biomass are exported^19^. In these low-input and nutrient-poor ecosystems, the available base cations are mainly derived from the dissolution of soil minerals and rocks. In this context, many of the soil microorganisms living in such conditions are likely facing nutritive stress, which probably determines their distribution and functioning.

The potential relationships between the nutrient availability and the taxonomic and functional structures of the soil bacterial communities have been reported in several studies considering soil chronosequences^20,21^, soil gradients^22–24^, soil profiles^25,26^, or the effect of inorganic fertilization with Ca or Mg in forest sites^27,28^. However, in most of these studies, the main soil factors considered were the pH and the availability of C, P or N forms and rarely the availability of base cations by themselves. Among the soil bacterial communities that may be the most impacted by variations in the availability of base cations are those involved in the dissolution of soil minerals (*e*.*g*., mineral weathering bacteria). Indeed, mineral weathering bacteria have been found in various nutrient-poor ecosystems such as deserts or forests^29,30^ and in different microbial habitats such as in the (mycor)-rhizosphere^29,31^, bulk soil^26^ or on the surface of minerals^20,32–37^. The frequency and effectiveness of mineral weathering bacteria were notably shown to vary following a mineral amendment^27,28^ or according to the physico-chemical properties of the mineral substrates^34,37^. In both cases, the conditions presenting the highest base cation availability (i.e., amended soil, highly weatherable minerals) were characterized by the lowest frequency of effective mineral weathering bacteria, suggesting a potential link between this functional group and nutrient availability. The ability to weather minerals is a functional trait observed in a wide range of bacterial genera^38^. It was proposed to be a functional adaptation to nutrient limiting environments^39^, making effective mineral weathering bacteria potentially more competitive in such conditions or stress tolerators according to the Grime definition^1^. However, a common feature of all these studies was the co-variation of the nutrient availability and pH, due to the *in situ* variations of the soil parameters^40,41^ and/or to the effect of the mineral amendment (*i*.*e*., liming^28^), limiting the conclusions. Indeed, pH is known as one of the main soil parameters determining the distribution of the soil bacterial communities^24^ and liming is used to rectify the acidity and fertility of the soil^42,43^.

In this context, our study aimed to understand how the availability of important base cations, such as potassium or magnesium, drives the taxonomic and functional distribution of the soil bacterial communities and especially the mineral weathering bacteria in forest soil. Such questioning is important in forestry, as liming is considered as an effective management practice to recover soil fertility and its use may be intensified to increase wood productivity. Consequently, it is important to know how such amendment will impact soil bacterial communities and their functioning. To do so, we considered a nutrient-poor soil (classified as Hyperdystric Cambisol) from the Montiers forest experimental site (France) characterized by a low concentration of base cations, limiting for the growth of trees for K and Mg^17^ and a high proportion of effective mineral weathering bacterial communities^41^. Calcium was not considered in our study, as it was not limiting in this soil^17^. Notably, the soil chemical analyses collected from this site and from other studies evidenced an enrichment of effective mineral weathering bacteria in soil conditions limited in potassium and magnesium compared to nutrient-rich conditions^41^. To disentangle the relative effect of the base cation availability from the effect of the pH on the soil bacterial communities, we developed a soil microcosm approach based on a fertilization with K or Mg. These nutrients were added to the soil in aqueous solution presenting the same pH as the pH of the soil considered. This experimental design allowed us to test the hypothesis that under nutrient-poor conditions (*i*.*e*., limiting in K and Mg), mineral weathering bacteria are competitive, while in nutrient-rich conditions (*i*.*e*., fertilized with K or Mg), they are not. Consequently, an increase in base cation availability is expected to directly impact mineral weathering bacteria and to reduce their frequency and effectiveness. As we did not know if the effect of the K or Mg fertilization on the soil bacterial communities was immediate, analyses were performed at different time points. During a 2-month period following K- or Mg-fertilization, the functional response of microbial communities through basal respiration (MicroResp) and carbon-substrate metabolization (Biolog EcoPlate) measurements was evaluated each 15 day-period. The soil chemical properties were measured to demonstrate the increase of available K or Mg in the soil as well as the stability of the pH. The mineral weathering potential of the bacterial communities was studied through a culture-dependent method. In addition, the taxonomic structure of bacterial communities was analysed by 16S rRNA amplicon-pyrosequencing. The same soil samples were also used for quantitative PCR analyses to quantify the total bacterial abundance as well as specific bacterial genera such as *Burkholderia*, *Collimonas* and *Pseudomonas*.

## Results

### Soil chemical analyses

Soil chemical analyses confirmed that the chemical parameters of the soil were modified according to the treatments applied (i.e., water only (Ct), water + Mg (Mg) or water + K (K) input) at each sampling time (Table 1). When considered overall, the K-fertilized treatment exhibited a significantly higher exchangeable K content (K: 0.23 ± 0.02 cmol+ kg^−1^) than the control and the Mg-fertilized treatments (control (Ct) and treatment fertilized with Mg: 0.12 ± 0.004 cmol+ kg^−1^). Similarly, the Mg-fertilized treatment exhibited a significantly higher exchangeable Mg content (Mg: 0.32 ± 0.05 cmol+ kg^−1^) compared to the control and the K-fertilized treatments (control (Ct): 0.07 ± 0.008 cmol+ kg^−1^ and treatment fertilized with K: 0.05 ± 0.007 cmol+ kg^−1^). Notably, the cationic exchange capacity (CEC) was not significantly modified by the fertilization treatments (P > 0.05). The other parameters remained unchanged compared to those in the control treatment, except for the exchangeable Al^3+^ or slight modifications at the T_3_ (i.e., 45 days) sampling time (Na, Mn). No significant time effect was observed for the other soil chemical characteristics (P > 0.05).

**Table 1:** Chemical characteristics of the soil samples collected among the three treatments (control, K and Mg fertilized) and the three sampling times (T0, T1 and T3).

Time	Treatment	K	Ca	Fe	Mg	Mn	Na	Al^3+^	H^+^	CEC	pH
		cmol+ kg^−1^ of soil									
T0	Ct	0.12	0.29	0.0029	0.077	0.21	0.067	2.25	0.14	3.17	4.65
T1	Ct	0.12a	0.29	0.0030	0.077a	0.20a	0.040	2.29a	0.13	3.15	4.66
	+K	0.28b	0.16	0.0023	0.047a	0.23b	0.038	2.19b	0.13	3.07	4.60
	+Mg	0.12a	0.21	0.0024	0.39b	0.23b	0.025	2.11c	0.12	3.20	4.56
Stat		**<0**.**0001**	0.23	0.29	**<0**.**0001**	**0**.**023**	0.89	**0**.**0004**	0.4	0.6	0.06
T3	Ct	0.12a	0.26	0.0024	0.07a	0.21a	0.003	2.29a	0.12	3.06	4.64
	+K	0.27b	0.17	0.0024	0.048a	0.23ab	0.043	2.20b	0.11	3.06	4.6
	+Mg	0.12a	0.26	0.0021	0.45b	0.25b	0.043	1.98c	0.10	3.29	4.59
Stat		**<0**.**0001**	0.49	0.60	**<0**.**0001**	**0**.**009**	0.22	**<0**.**0001**	0.06	0.13	0.11

The quantities of exchangeable elements and the CEC are expressed in cmol of charge per kg of soil. CEC: cation exchange capacity. The data presented are mean values of 3 replicates. Different letters (a, b, c) indicated significant differences between treatments calculated for the sampling times T1 and T3 separately, according to a one-factor ANOVA analysis followed by a Tukey test (P < 0.05). Significantly higher values were indicated in bold. For legibility reasons, the standard error values are not presented.

### Carbon substrate metabolization ability

When all the time points were combined and regardless the treatment, overall higher metabolization potentials were observed at T_1_ (i.e., 15 days after the fertilization) for carboxylic acids (T1 > T2 = T3 = T4; P = 0.003) and on the contrary lower metabolization potentials at T_1_ for polymers and miscellaneous substrates (T1 < T2 = T3 = T4; P_polymers_ = and P_miscellaneous_ = 0.0004). When the treatments were considered, a significant effect was observed for the Mg fertilization compared to the control for the polymers (P = 0.003; Mg ≥ K ≥ Ct) and the carboxylic acids (P = 0.0002; Ct ≥ K ≥ Mg).

When each time point (i.e. T1 to T4) was considered independently, our analyses revealed a significant effect of Mg fertilization on the absorbance measures (P < 0.05; Table S1). For the carboxylic acids, a significant decrease was observed in the Mg and K fertilized treatments compared to the control treatment at T2 (and a trend at T3 and T4; Table S1). For polymers, a significant increase was observed in the Mg fertilized treatment compared to the control treatment at T2, T3 (and a trend at T4; Table S1).

### Respiration profiles

The MicroResp analyses revealed a decreasing respiration rate in the control treatment between T_0_ and the other sampling times (Fig. S1; Resp_T0_: 2.5 µg CO2/gr of soil (Dw)/hr; Resp_T1_: 1.77; Resp_T2_: 1.74; Resp_T3_: 1.64; Resp_T4_:1.64; P < 0.0001). The same MicrResp analyses also showed both treatment and time effects according to a 2-factor (time, treatment) ANOVA (P = 0.02). When analysed globally, the addition of K and Mg induced a decrease in microbial respiration compared to that of the control treatment (Resp_K_: 1.51 ± 0.03; Resp_Mg_: 1.40 ± 0.01 vs Resp_Conrtol_: 1.70 ± 0.03, P < 0.05), but this effect decreased with time. At T_1_ (i.e., 15 days), the addition of Mg caused a significant decrease compared to the control treatment (Resp_K_: 1.70; Resp_Mg_: 1.43; Resp_Control_: 1.77; P = 0.005), but the addition of K did not. At T_2_ (30 days), both K and Mg treatments allowed a significant decrease compared to the control treatment (Resp_K_: 1.35; Resp_Mg_: 1.34; Resp_Control_: 1.74; P < 0.0005). No significant effect of K and Mg addition was observed at T_3_ (i.e., 45 days) and T_4_ (i.e., 60 days) sampling times compared to the control, although a decreasing respiration rate was observed.

### Bacterial densities

Dilutions and plate counting revealed relatively similar culturable bacterial densities between treatments and sampling times, ranging from 1.15 × 10^6^ to 3.47 × 10^6^ (expressed as CFU /gram of soil; Table 2).

**Table 2:** Bacterial quantification and ratio analyses.

Treatment	Culture	qPCR				(Ratio)		
	Total bacteria	Total bacteria	*Burkholderia*	*Collimonas*	*Pseudomonas*	T/B	T/C	T/P
T1-Ct	6.50a	7.94a	6.06a	6.03d	6.03a	76.63b	86.80a	81.93a
T1 + K	6.38a	7.77a	5.49b	5.47ab	5.56a	191.67a	204.67ab	234.97a
T1 + Mg	6.11a	7.68a	5.52b	5.31abc	5.38b	150.27ab	249.67ab	207.33a
T3-Ct	6.42a	7.59a	5.74b	5.71ad	5.44b	80.10b	79.83a	150.67a
T3 + K	6.06a	7.75a	5.58b	5.07bc	5.40b	150.00ab	494.33ab	224.00a
T3 + Mg	6.11a	7.74a	5.51b	4.93c	5.52b	170.67a	717.33b	173.00a

Quantification done by dilution/plating or quantitative PCR are expressed as Log[cfu g^−1^ of soil] and Log[16S rRNA gene copies g^−1^ of soil], respectively. The data presented are mean values of 3 replicates. Ratio analyses (i.e., T/B: ratio Total bacteria/*Burkholderia*; T/P: ratio Total bacteria/*Pseudomonas*; T/C: ratio Total bacteria/*Collimonas*) have been done on the non-log transformed data. Treatments presenting different letters (a,b,c,d) are significantly different according to a one-factor ANOVA and Tukey post-hoc test (P < 0.05). Treatments are presented as follows: Ct, control; +Mg, Mg fertilized and +k, K fertilized. For legibility reasons, the standard error values are not presented.

Determination of the total bacterial densities by qPCR did not reveal any treatment or time effects, with densities ranging from 3.89 × 10^7^ to 8.71 × 10^7^ (expressed as 16S rRNA gene copies/gram of soil]. Quantification of *Burkholderia* revealed a decrease between the control and fertilized (K and Mg) treatments at T_1_ and T_3_; however, this was only significant at the T_1_ sampling time (Ct-T1: 1.15 × 10^6^ vs 3.09 × 10^6^ and 3.31 × 10^6^ for K and Mg, respectively; P < 0.0005). Quantification of *Collimonas* revealed a decrease between the control and fertilized (K and Mg) treatments at T_1_ and T_3_; however, this was only significant at the T_1_ sampling time (Ct-T1: 1.07 × 10^6^ vs 2.95 × 10^5^ and 2.04 × 10^5^ for K and Mg, respectively; P < 0.0001). Quantification of *Pseudomonas* revealed a decrease between the control and Mg-fertilized treatment only at the T_1_ sampling time (Ct-T_1_: 1.07 × 10^6^ vs 2.4 × 10^5^ for Mg; P < 0.05). The relative decrease of the abundance of *Burkholderia* and *Collimonas* according to the K and Mg fertilizations was confirmed by a global analysis of the ratios (total bacteria/*Burkholderia*, P = 0.003*;* total bacteria/*Collimonas*, P = 0.008), but no significant effect was observed for *Pseudomonas* (Table 2).

### Bacterial diversity and community profiles

Analyses of the total 16S rRNA sequence dataset revealed globally high coverage according to the coverage index, ranging from 84 ± 0.01% in the control treatment at T_1_ (Ct_T_1_) to 89 ± 0% in the control and K-fertilized treatments at T_2_ and T_4_ (Table S2). Analysis of the α diversity revealed no difference between the treatments and sampling times for the Shannon indices (average: 3.82 ± 0.07) and the number of observed OTUs (average S_obs_: 110 ± 3). A sampling time effect was observed for the Chao and Simpson indices with a higher diversity in the T_1_ (Simpson: 36.80 ± 3.72; Chao: 226.40 ± 13.06) than in the T_3_ (Simpson: 16.66 ± 1.91; Chao: 176.66 ± 9.30) sampling time (P = 0.0009 and P = 0.0144, respectively). Concerning the community structure, no treatment and sampling time effect was observed according to PermANOVA analyses performed on the OTU table (P > 0.05).

### Taxonomic composition of bacterial communities

A total of 13 phyla (55% of the sequences), 25 classes (53%), 41 orders (52%), 49 families (46%) and 53 genera (14%) were identified in our 16S rRNA sequence dataset. The first three phyla of the dataset represented more than 50% of the total sequences and were affiliated with Acidobacteria (30%), Proteobacteria (17%; Alphaproteobacteria: 9%, Gammaproteobacteria: 4%, Betaproteobacteria: 3% and Deltaproteobacteria: 1%; Fig. S2) and Actinobacteria (3.5%). The other phyla, Saccharibacteria, Chlamydiae, Bacteroidetes, Gemmatimonadetes, Verrucomicrobia, Nitrospirae, Elusimicrobia, Firmicutes and Armatimonadetes, represented less than 1% of the total dataset each. The top 10 most represented genera in the 16S rRNA sequences were *Burkholderia* (1.4%), *Acidothermus* (1.3%), *Collimonas* (0.3%), *Rhizomicrobium* (0.3%), *Variibacter* (0.2%), *Streptacidiphilus* (0.1%), *Cupriavidus* (0.1%), *Dokdonella* (0.1%), *Nitrospira* (0.08%) and *Roseiarcus* (0.07%).

When each sampling time was considered independently, significant differences were observed between the different treatments (Table S3). Betaproteobacteria were significantly more abundant in the control treatment at T_2_ (i.e., 30 days) than in the K-fertilized treatment. This difference was partly explained by a higher proportion of sequences assigned to Burkholderiales in the control treatment at T_1_ and T_2_ compared to the K-fertilized treatment. However, this effect was not observed at the genus level. At the OTU level, significant differences were observed among treatments with i) the OTU0001 enriched in K-fertilized treatment compared to the control treatment (unclassified bacteria) and ii) the OTU0002 enriched in the control treatment compared to the K-fertilized treatment (unclassified bacteria).

### Mineral weathering potential of bacterial strains

**Figure 1:**
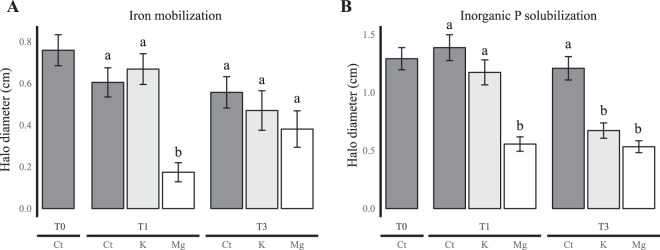
Relative mineral weathering effectiveness of the bacteria according to the treatment (control, K fertilized and Mg fertilized) and the sampling time (T0, T1 and T3). (**A**) Relative effectiveness at mobilizing iron and (**B**) Relative effectiveness at solubilizing inorganic phosphorus. For each bar, the data presented are mean +/− Standard deviation. The error bars indicate standard deviations. Lowercase letters (a or b) indicate significant differences between treatments in one sampling time. Statistics were obtained according to a one- factor ANOVA followed by a Tukey test (P < 0.05). A total of 412 and 357 bacterial strains have been tested on the TCP and CAS media, respectively. Each strain was tested with 3 replicates.

**Figure 2:**
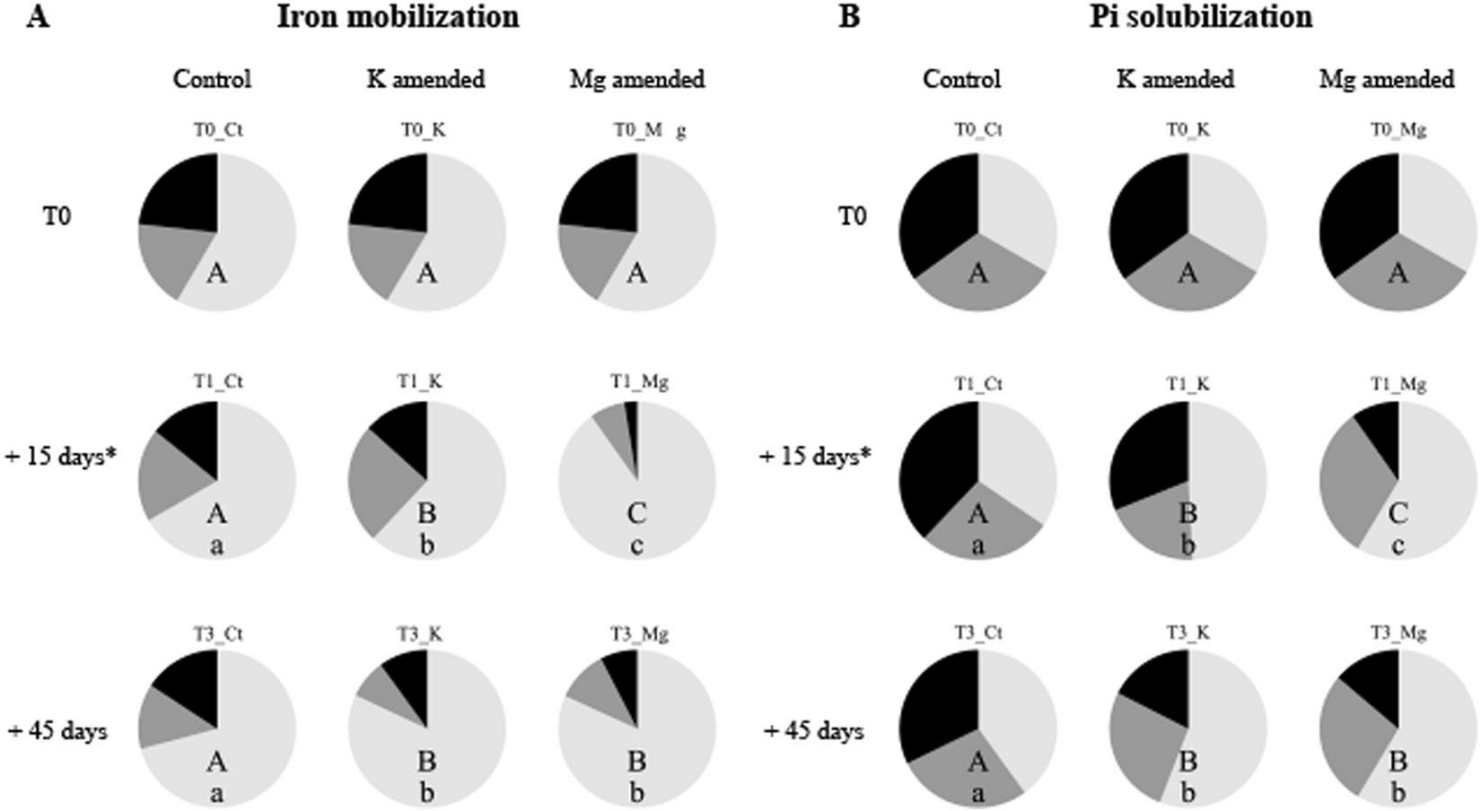
Distribution in classes of mineral weathering effectiveness of the bacteria depending on the treatment (control, K fertilized and Mg fertilized) and on the sampling time (T0, T1 and T3). (**A**) Iron mobilizing ability and (**B**) Inorganic phosphorus solubilizing ability. For each bioassay, determination of the class of effectiveness was done depending on the median (M) of the discoloration zones of the positive strains (black: strong activity, discoloration zone > M; grey: moderate activity, discoloration zone < M; white: no degradation activity). Capital letters (**A**,**B** or **C**) indicate significant differences between the sampling times, within one treatment. Lowercase letters (a,b of c) indicate significant differences between the treatments, within one sampling time. Statistics were obtained according to a Chi2 test (P < 0.05). A total of 412 and 357 bacterial strains have been tested on the TCP and CAS media, respectively. Each strain was tested with 3 replicates.

The functional screenings of the bacterial strains isolated from the different treatments highlighted a global negative impact of fertilization (Mg or K) on the relative distribution and effectiveness of the mineral weathering (MW) bacteria (Figs 1 and 2; P < 0.002).

Notably, the Mg-fertilization treatment had the strongest effect on the ability to mobilize iron (CAS; Fig. 1A) and to solubilize inorganic phosphorus (TCP; Fig. 1B). Indeed, in this treatment, the average MW effectiveness of the bacteria passed from T_0_ (T_0__TCP = 1.29 ± 0.10 and T0_CAS = 0.76 ± 0.07 cm of haloes) to a significantly lower average MW effectiveness at T_1_ (T_1__TCP = 0.56 ± 0.06 and T_1__CAS = 0.17 ± 0.05 cm of haloes) (P < 0.0001). The same trend was observed at T_3_ but was only significant for the ability to solubilize inorganic phosphorus. A significant effect of Mg fertilization was also observed on the relative distribution of the effective MW bacteria. This frequency significantly decreased from T_0_ (T_0__TCP: T_0_ = 35% and T_0__CAS = 23%) to T_1_ (TCP_T_1+Mg_ = 10% and CAS_T_1+Mg_ = 3%; both P < 0.0001, Fig. 2). The same trend was observed at T3.

For the K-fertilized treatment, a decrease in MW effectiveness was also observed, but later than the effect observed in the Mg-fertilized treatment (Fig. 1). Indeed, a significant reduction of the MW effectiveness occurred only between T_1_ and T_3_ [(T_1__CAS = 0.67 ± 0.07 cm and T_3__CAS = 0.47 ± 0.09 cm (Fig. 1A); P = 0.037; T_1__TCP = 1.17 ± 0.11 cm and T_3__TCP = 0.67 ± 0.07 cm of haloes (Fig. 1B); P < 0.0001)]. A significant effect of K fertilization was also observed on the relative distribution of the effective MW bacteria. This effect was observed between each sampling time for the ability to solubilize inorganic phosphorous (T_0__TCP = 35%, T_1__TCP = 31% and T_3__TCP = 18%) and to mobilize iron (T_0__CAS = 23%, T_1__CAS = 13% and T_3__CAS = 10%) (P < 0.0001; Fig. 2). Notably, such a time effect was not observed on the MW effectiveness and the frequency of mineral weathering bacteria in the control treatment (P > 0.05).

### Taxonomic affiliation of the bacterial strains and link with function

The taxonomic identification of the bacterial strains by the partial sequencing of the 16S rRNA gene showed that they belonged to the Proteobacteria [45%; Betaproteobacteria (28%), Gammaproteobacteria (5%) and Alphaproteobacteria (1%)], Firmicutes (34%) and Actinobacteria (21%)] phyla. The dominant genera identified were *Bacillus* (25%), *Collimonas* (18%) and *Arthrobacter* (18%), *Paenibacillus* (16%) and *Burkholderia* (8%).

Regarding the distribution of these genera according to the treatments applied in our study, our analyses revealed a significant effect of both fertilization treatment and sampling time on the taxonomic structure of the culturable representatives. Notably, the control treatment was dominated by bacteria belonging to Proteobacteria regardless of the sampling time considered and particularly by the *Collimonas* genus (T_0_: 28% of the collection). In contrast, the proportions of Proteobacteria and *Collimonas* strongly decreased with time in both fertilized treatments (after 45 days (T_3_): *Collimonas* = 3%; Chi^2^: P < 0.0001). In parallel, the proportion of bacterial strains affiliated with Firmicutes, particularly to the *Bacillus* genus, increased in the fertilized treatments at T_3_ (37%; P = 0.004), while it remained lower in the control treatment regardless of the sampling time (15%).

**Figure 3:**
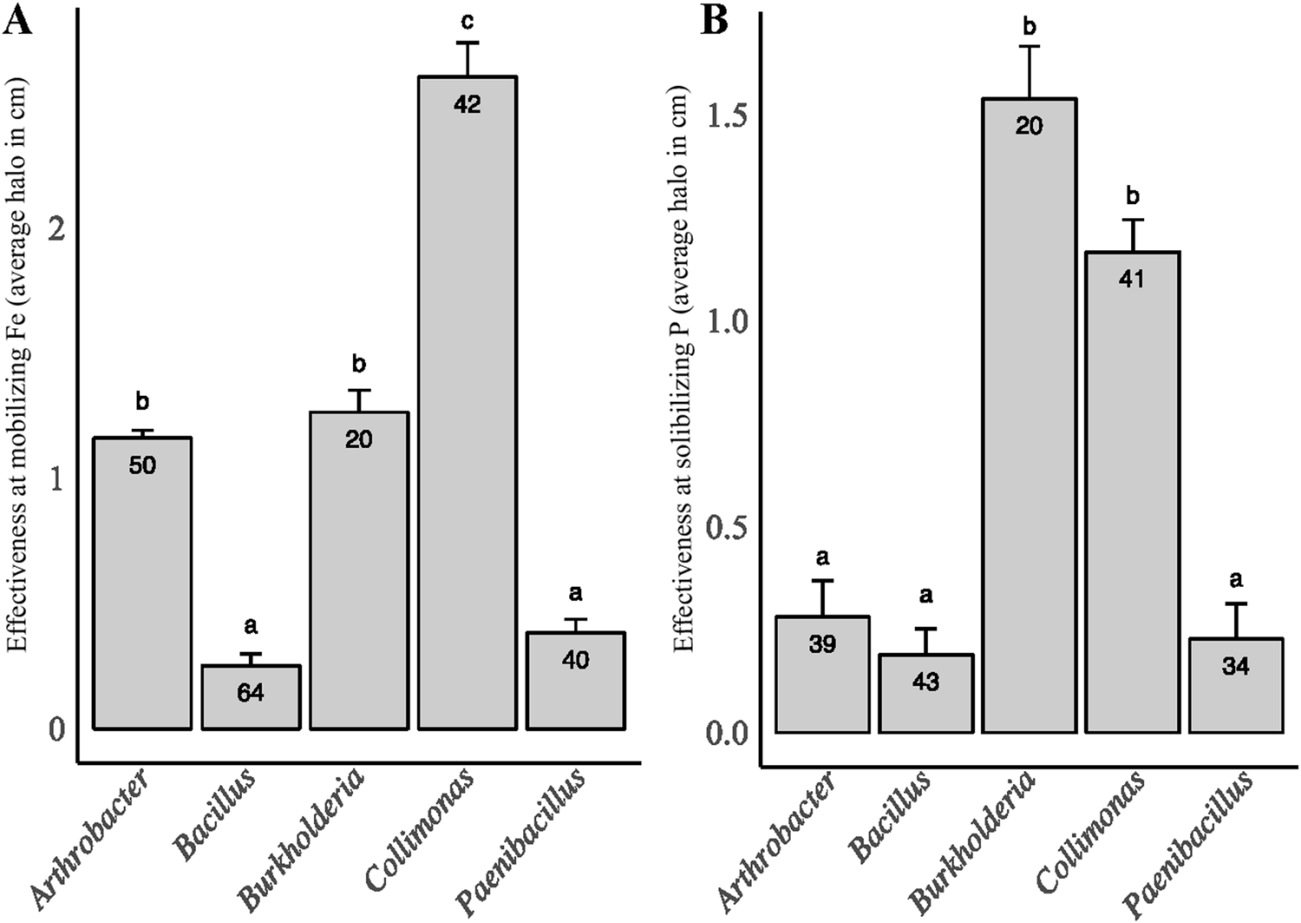
Relationship between mineral weathering effectiveness and taxonomic affiliation of the bacteria. For each bacterial genus represented by a minimum of 10 isolates, the relative effectiveness to: (**A**) mobilize iron and (**B**) solubilize inorganic phosphorus is presented, whatever the origin of the bacterial isolates. For each bar, the data presented are mean +/− Standard deviation. The error bars indicate standard deviations. Lowercase letters (a–c or d) indicate significant differences between genus effectiveness. Statistics were obtained according to a one-way ANOVA followed by a Tukey test (P < 0.05). The number of bacterial isolates tested per genus is presented into the bars.

Analysis of the potential relationship between taxonomy and functional ability revealed that the members of the genera *Burkholderia* and *Collimona*s exhibited the strongest MW effectiveness regardless of the bioassay considered (i.e., mobilization of iron and solubilization of inorganic phosphorus) compared to the other genera (Fig. 3). The members of the genus *Arthrobacter* were relatively effective at mobilizing iron. Notably, ca. 80% of the representatives of these two genera belonged to the bacterial groups that were very effective at mobilizing iron. For the ability to solubilize inorganic phosphorous, 80% of the *Collimonas* strains belonged to the most effective class, while *Burkholderia* (80%), *Arthrobacter* (96%) and the remaining *Collimonas* strains exhibited intermediate effectiveness. In contrast, members of the *Bacillus* and *Paenibacillus* genera exhibited very low mineral weathering effectiveness in both bioassays. Notably, our analyses also revealed no significant difference in effectiveness within a genus between treatments or sampling times.

Comparison of the overlapping portion of the 16S gene sequences between the 454-pyrosequences and Sanger sequences of the bacterial strains revealed a relatively good overlap between the two datasets (Table S4). A total of 87% of the bacterial strains from the collection exhibited more than 97% sequence similarity with the pyrosequences, while only 3.5% of the pyrosequences exhibited high similarity with the Sanger sequences from the bacterial collection. Notably, many Sanger sequences of our bacterial collection presented more than 97% sequence similarity with highly represented OTUs (i.e., OTU14, *Burkholderia*; OTU19, *Rhodanobacter*; OTU55, *Collimonas*) or poorly represented OTUs (OTU776 and OTU829, *Paenibacillus*; OTU866, *Rhizobium*).

## Discussion

Using a combination of culture-dependent and -independent methods associated with a microcosm approach, we tested the hypothesis that an increase in the concentration of a single base cation in a soil naturally depleted in this element can modify the taxonomic and functional structures of the soil bacterial communities. The resource manipulation we applied in our study to nutrient-poor soil was determined to mimic the nutrient-rich conditions occurring in the nutrient-rich soil of the Montiers toposequence^41^. In this sense, our fertilization reached the quantity of available K (i.e., 0.28 cmol+ kg^−1^ of soil) and half of the quantity of available Mg (i.e., 0.81 cmol+ kg^−1^ of soil) measured in the nutrient-rich soil of the Montiers toposequence^41^. Consequently, the fertilization applied was low and corresponded to an increase of 2 and 5 times the available K and Mg in the manipulated nutrient-poor soil. This approach allowed us to highlight how the diversity, composition and function of the bacterial communities were impacted by an increase in the availability of base cations. To our knowledge, this study represents the first experimental demonstration of the effect of the availability of base cations and of its short-term effect on the taxonomic and functional structure of soil bacterial communities. Notably, our culture-dependent approach coupled with qPCR highlighted that the modification of the functional structure observed, which corresponded to a decrease of the frequency of effective mineral weathering bacteria, was related to a decrease in specific taxa.

Compared to other edaphic parameters, such as pH or variations of the concentration of organic compounds, few studies have demonstrated a potential impact of the availability of base cations, such as Mg, K or Ca, on the soil bacterial communities^40,44,45^. The role of these nutrients has mainly been investigated in the context of fertilization (i.e., NPK or dolomite amendments^45,46^) or as potential toxic elements^47^. Consequently, little is known about their relative effects on soil microorganisms. This discrepancy is partly because most of the environmental studies mainly focused on the C, N or P cycles, as many soil parameters are linked, co-vary and have low concentrations of the base cations in comparison with the amounts of C and N. Indeed, base cations such as Mg, K or Ca usually present concentrations 100 to 1000 times lower than C. However, these cations are essential nutritive elements whose depletion strongly affects plant growth, such as in forest ecosystems developed on nutrient-poor soils^16,48–50^. These cations originated from the recycling of the cations contained in dead organic matter or from the weathering of soil minerals. In the soil considered in our study, which was sampled from the organic-mineral horizon, the base cations were mainly derived from the weathering of soil minerals^17^. In both organic matter decomposition and mineral weathering processes, microorganisms have been identified as key actors^51^. Consequently, an increase or depletion of the concentrations of base cations should directly impact their distribution and function.

The functional analyses performed during the 2-month incubation period in our study showed the impact of resource manipulation on the functional structure of the soil microbial communities. Notably, global respiration and catabolic activities appeared to be affected, but only at a low level. Indeed, both activities appeared slightly decreased in the fertilized treatments with a higher effect of Mg, suggesting a functional switch or a potential toxic effect of the cations added to the microbial communities involved in OM decomposition^47^. However, no effect was observable on the catabolic diversity (i.e., Biolog, Shannon index values) nor on the total bacterial quantifications performed both by dilution/plating and qPCR. These findings indicate that fertilization with K or Mg has a negligible effect on the global development of the soil bacterial communities compared to those in the control treatment, excluding the toxicity hypothesis. The few differences observed between the treatments may be related to the two functions measured (i.e., respiration and catabolic potentials). In contrast, the mineral weathering ability of the soil bacterial communities measured using bioassays highlighted a strong decrease of this functional group in the amended treatments, with a higher effect of Mg than K compared to the control treatment. Together, these results suggest an adaptation of the soil microbial communities to an increase in nutritive resources. While the mineral weathering ability seems to be advantageous for the bacteria with this functional trait in nutrient-poor conditions, it is not advantageous in nutrient-rich conditions, and this group is outcompeted.

The global analysis performed on the structure of the soil bacterial communities based on 16S rRNA amplicon pyrosequencing data did not reveal significant variations between the different treatments. Noticeably, the community composition and structure obtained in our microcosm-based study (*i*.*e*., dominance of Acidobacteria and Proteobacteria and high proportion of unclassified bacteria) appeared very similar to those obtained from the same soil, but from a field experiment in a previous study^40^. A BLASTN analysis revealed that most of the OTUs generated in our study, including the unclassified_bacteria, presented a high sequence homology (99 to 100% identity) with OTU sequences generated by Colin *et al*.^40^ (SRA based comparison). The absence of significant variations according to the treatments applied in our study suggests a low effect of the cation input on the taxonomic structure and composition of the soil microbiome. However, a detailed analysis revealed an increase in the relative abundance of pyrosequences assigned to Actinobacteria (corresponding mainly to *Arthrobacter*) and a decrease in the relative abundance of pyrosequences assigned to Betaproteobacteria (Burkholderiales) in the fertilized treatments compared to the control. Notably, the relative abundance of 16S rRNA pyrosequences assigned to *Burkholderia* (*de novo Paraburkholderia* and *Caballeronia*) and *Collimonas*, two genera known for their mineral weathering effectiveness^27,52,53^ decreased in the fertilized treatments (+K, +Mg) compared to that in the control. Effective mineral weathering bacteria affiliated to other genera, such as *Arthrobacter*, have been previously described^20,26^ but presented a low mineral weathering potential in our study. The decrease of Burkholderiales was confirmed by the genus-specific qPCR analyses, which showed a significant decrease of *Burkholderia* and *Collimonas* in the fertilized samples only. This effect was not observed for the qPCR analyses targeting *Pseudomonas*. Together, our new results highlight that K or Mg fertilization minimally affects the total density and structure of the soil bacterial communities, while specific bacterial genera such as *Arthrobacter*, *Burkholderia* and *Collimonas* are strongly affected.

Representatives of the Burkholderiales order are known to represent an important proportion of the taxa assigned from culture-dependent and culture-independent analyses in acidic and nutrient-poor forest soils. *Burkholderia* and *Collimonas* strains have been isolated, especially in the (mycor-)rhizosphere^54^ and in the mineralosphere^34,37^. Notably, in the mineralosphere, these taxa appeared significantly enriched on the surfaces of the less-weatherable minerals^34^, suggesting a link between these taxa and the mineral chemistry and/or the nutrient availability. Interestingly, Kelly *et al*.^55^, analysing the active part of the microbiome, also revealed a variation of the proportion of *Burkholderiales* in the presence of minerals and according to the physico-chemical properties of the mineral. In our study, the relative abundance of representatives of the *Burkholderia* and *Collimonas* genera decreased in the fertilized treatments compared to those in the control, suggesting that these genera are non-adapted or less competitive to an increase in nutrient availability. Culturable representatives of these genera are heterotrophs and have been proposed to be classified as copiotrophs^56,57^. Copiotrophs are organisms adapted to live in nutrient-rich environments, particularly rich in carbon. Their physiological requirements are usually superior to oligotrophs organisms which on the contrary live in nutrient-poor environments and require limited resources to grow^56,57^. However, our findings highlight that some of these Burkholderiales-related taxa (i.e., *Burkholderia* and *Collimonas*) have oligotrophic behaviour based on their ability to inhabit nutrient-poor environments and their effectiveness in mineral weathering^37,53,58,59^. Moreover, Lepleux *et al*.^37^ demonstrated that phylogenetically related *Burkholderia* strains isolated from the same soil but from different habitats (bulk soil vs mineralosphere) harboured different metabolic potentials and mineral weathering potentials. Indeed, the *Burkholderia* strains isolated from the mineral surfaces consumed few carbon substrates with a low effectiveness compared to those of the bulk soil, while they belonged to the same species. Representatives of the *Collimonas* genus have been mainly reported in nutrient-poor environments (soil, dune, tundra)^53,58,60^. Consequently, those results fit very well with Ho *et al*.^2^, who proposed that different functional strategies may exist in the same bacterial order, family or even genus.

## Conclusion

Through the resource manipulation performed, this work demonstrated for the first time that an increase of base cation (*i*.*e*., K and Mg) concentration in the soil more strongly affects the functional structure of the soil bacterial communities than the taxonomic structure. This difference between the total taxonomic structure and the functional structure (*i*.*e*., mineral weathering ability) of the bacterial communities may be explained by the fact that an important proportion of the soil microbiome is driven by the availability of organic nutrients (*i*.*e*., C and N), while a smaller proportion is driven by the availability of inorganic nutrients (*i*.*e*., Ca, K and Mg). An important point in our study is that the soil pH was not modified by the resource manipulation, making a real analysis of the effect of cation availability on the soil microbiome possible. Notably, both the culture-dependent and qPCR analyses revealed no effect of fertilization on the total densities of the bacterial communities during our experiment but a significant decrease in the densities of the *Burkholderia* and *Collimonas* populations. Interestingly, the *Burkholderia* and *Collimonas* strains isolated in our study harboured a greater effectiveness to weather minerals, confirming previous findings obtained for these two taxa. Together, our results show that under nutrient-poor conditions (*i*.*e*., non-fertilized soil), these two taxa were dominant, while they were less competitive in nutrient-rich (+K or +Mg) conditions. This finding fits very well with the definition by Grime, which considers organisms capable of developing in limiting conditions as stress tolerators. Based on this definition, our results and previous findings obtained for *Burkholderia* and *Collimonas* strains isolated in different geographical locations in forest soils, we proposed to consider them as oligotrophic and effective mineral weathering taxa. These results also bring new perspectives to soil microbial ecology and land use, as the effect of fertilization with base cations on the functional diversity has been rarely considered.

## Materials and Methods

### Site description and soil sampling

In January 2017, soil samples were collected from the Montiers forest experimental site located in the Meuse department (northeastern France). This long-term observatory (LTO) is part of AnaEE-France (Analyses et Expérimentations sur les Ecosystèmes - France). It is co-managed by the ANDRA [French national radioactive waste management agency; Permanent Environment Observatory (OPE)] and the INRA (UMR1138 Unit; http://www.nancy.inra.fr/en/Outils-et-Ressources/montiers-ecosystem-research). The site is dominated by a homogeneous land cover of European beech trees of the even-aged stand (*Fagus sylvatica L*.*;* 50 years old in 2010) and is characterized by a Jurassic limestone (Tithonian) layer overlaid by acid detrital sediments from the lower Cretaceous (Valanginian). The site is characterized by a soil toposequence covering different soil types: from high-nutritive Cambisol Calcaric to nutrient-poor Cambisol Hyperdystric, with an intermediate Eutric Cambisol^17,41,44,61^. In this study, sampling was performed in Cambisol Hyperdystric soil, which is the most nutrient-poor soil type of the toposequence. The soil was sampled from the same plot after removing leaf litter. The 0–5 cm soil layer was separated, and only the 5–20-cm soil horizon was transported back to the lab. This soil horizon corresponds to the organo-mineral horizon of the soil profile and to the horizon where most of the fine roots of the trees are located. Soil samples were collected using sterile tools to avoid contamination and were returned to the laboratory immediately. All the soil samples were then mixed to homogenize and sieved by a 4 mm mesh. Then, they were stored at 4 °C overnight before the beginning of the experiment.

### Microcosm experimental design

A microcosm experiment was conducted to test the relative impact of an increase in nutrient availability on the functional and taxonomic structures of the soil bacterial communities. To do so, we considered two important base cations (magnesium and potassium). These nutrients were selected as previous experiments performed on the Montiers site noted the potential role of magnesium and potassium as limiting base cations for the growth of trees in the Cambisol Hyperdystric soil compared to the Cambisol Calcaric soil^17^ and because these two nutrients are important cations for tree growth in nutrient-poor and acidic forests^41^. Moreover, according to the fertility norms established for forest soils^62^, the fertility in K is low in Cambisol Hyperdystric soil, and the fertility in Mg is moderate and at the limit of the norm^17^. The calcium was not considered in our study, as it is not limiting at the site considered as it is developed on limestone rich in calcium. The microcosms were prepared the day following the field sampling so that soil was fresh. The soil was separated into three soil subsamples to allow the application of the different treatments in the soil: (i) control (water), (ii) water + magnesium, and (iii) water + potassium. The concentrations of magnesium and potassium used to fertilize the Cambisol Hyperdystric soil were chosen to reach the same concentrations of available Mg and K in the microcosms as the concentrations of these elements measured in the most nutrient-rich soil type of the toposequence of Montiers (*i*.*e*., the Calcaric Cambisol; ref.^41^ [Table 1]). The different treatments considered in our microcosm experiments were (i) K fertilization (KCl, 158.7 mg kg^−1^ dw (dry weight) soil), (ii) Mg fertilization (MgCl_2_, 701.6 mg kg^−1^ dw soil), and (iii) distilled water (control). Distilled water was used to prepare the different solutions. Each solution was adjusted to the same pH (4.6), which was the pH of the Cambisol Hyperdystric soil sampled, to avoid any effects due to the pH of the solutions added to the microcosms. Treatments were applied in one pulse at the beginning of the experiment. The moisture of the microcosms was adjusted according to the field soil moisture (35% of the water holding capacity (WHC)), and the soil water content was adjusted throughout the experiment with sterile distilled water. After the addition of the control (water), K and Mg solutions, each of the soil subsamples was mixed and divided into 20 microcosms for the control treatment (including 4 T0 microcosms), 16 microcosms for the K treatment and 16 microcosms for the Mg treatment, giving a total of 52 independent microcosms. The microcosms used consisted of small pots (40 mL, sterile) filled with 27 g of Cambisol Hyperdystric soil (fresh weight) fertilized with the different solutions described above. The microcosms were incubated in a dark experimental chamber at 21 °C for 60 days.

### Sample collection

The experiments were run over 60 days, and soil samples were collected at different time points: i) just after the application of the treatment (T_0_), ii) after 15 days (T_1_), iii) after 30 days (T_2_), iv) after 45 (T_3_) and v) after 60 days (T_4_). At each sampling time, four microcosms from each treatment were selected to perform the different analyses described below. For each microcosm, the soil collected (27 g) was homogenized before any analysis. A total of 1.5 g of the soil was used to determine the global respiration using the MicroResp system. A total of 5 g of the soil was suspended in 25 mL of sterile distilled water and vortexed twice for 1 min. This soil suspension was diluted at 1/20 to determine the global metabolic potential on Biolog EcoPlates. In parallel, a volume of 0.5 mL of the undiluted soil suspension was used to perform culture-dependent bacterial analyses. A total of 1 g of the soil was conserved at -20 °C for DNA-based analyses. Finally, 5 g of the soil was dried at 35 °C and sieved at 2 mm for chemical analyses.

### Soil chemical analyses

The cation exchange capacity (CEC) was determined using the cobaltihexamine method^63^, which is based on the titration of the cobaltihexamine chloride soil extract at 472 nm compared to a reference 0.05 N cobaltihexamine chloride extract. The pH was determined by the water method using a soil/water ratio of 1:5 (w/v). Exchangeable cations (Ca, Mg, Na, K, Fe, Mn and Al) and H^+^ were extracted using cobaltihexamine and determined by inductively coupled plasma spectrometry-atomic emission spectrometry (ICP-AES) for cations and by potentiometric measurement using 0.05 M KOH for protons and aluminium^64^. The chemical analyses were performed on the four replicates of each treatment (potassium, magnesium or water) at the T_0_, T_1_ and T_3_ sampling times.

### Substrate metabolization assay

The ability of microbial communities to metabolize various carbon substrates was assessed at each sampling time and for each treatment through the EcoPlates (Biolog®; Hayward, CA95545 U.S.A), which contain 31 different carbon substrates (pyruvic acid methyl ester;tween 40; tween 80; alpha-cyclodextrin; glycogen; D-cellubiose; alpha-D-Lactose; beta-methyl-D-glucoside; D-xylose; i-Erythritol; D-Mannitol; N-Acetyl-D-Glucosamine; D-Glucosaminidic acid; Glucose 1 phosphate; Glycerol phosphate; D-galactonique acid gamma-lactone; D-galacturonic acid; 2-Hydroxy-benzoic acid; 4-Hydroxy-benzoic acid; Hydroxybutiric acid; Itaconic acid; ketobutyric acid; malic acid; arginine; asparagine; phenylalanine; serine; threonine; glutamic acid; phenylethylamine; putrescine). A volume of 150 µL of the 1/20 diluted soil suspension described above was loaded in each well of a Biolog microplate and incubated at 25 °C for 48 h. For the soil considered 48 h of incubation was enough, saturation was obtained after this period. Colour development was measured at 595 nm with an automatic microplate reader (Bio-Rad, model iMark). The absorbance data were corrected by subtracting the blank, and negative absorbance values were set to zero. The data were expressed as average well color development (AWCD)^65^. The measures were performed on three replicates biological.

### Respiration profiles

The basal soil microbial respiration was measured by the MicroResp method^66^ at each sampling time and for each treatment. The measurements were performed on the four replicates. For each replicate, 4 deep-wells of the MicroResp microplate were filled with 0.3 g of soil. Simultaneously, a CO_2_-detection microplate was filled with a pH indicator gel (3% agar, 2.5 mM NaHCO_3_, 150 mM KCl, and 12.5 µg mL^−1^ of cresol red). The deep-well microplate was placed face-to-face with the CO_2_-detection microplate, and both were firmly sealed together with a silicone rubber gasket following the manufacturer’s instructions^64^. After 6 h of incubation in the dark at 25 °C, the pH change was visible through a colour shift of the indicator gel and was measured at 570 nm with an automatic microplate reader (BioRad, model iMark). The rate of CO_2_ (µg CO_2_/gr of dw soil/hr) produced was calculated according to Campbell *et al*.^66^.

### Bacterial collection

For each treatment, bacterial collections were performed considering three replicates. For each replicate, 5 grams of soil was sampled and suspended in 25 mL of sterile distilled water. A volume of 0.2 mL of the pure soil suspension was serially diluted in sterile distilled water and spread onto a 1/10 diluted tryptic soy agar (TSA) medium (tryptic soy broth from Difco, 3 g L^−1^ and agar, 15 g L^−1^) supplemented with 100 µg L^−1^ cycloheximide (Sigma; final concentration). The Petri dishes were incubated at 25 °C for 5 days. Colonies were counted after the incubation period and expressed as colony forming units (CFUs) g^−1^ of soil (dry weight). For each of the three biological replicates and considering the same dilution for all the treatments, all the colonies were collected. In our study, it corresponded to ca 20 colonies per replicate for a total of 60 bacterial strains per treatment and sampling time. The bacterial isolates were purified by three successive platings on 1/10 diluted TSA and then cryopreserved at −80 °C in 20% glycerol. A total of 420 bacterial strains were isolated. All the bacterial isolates used in this study were then cultivated on 1/10 diluted TSA at 25 °C for 72 h.

### Mineral weathering potential of bacterial isolates

The mineral weathering potential, meaning the ability to mobilize inorganic nutrients of each bacterial isolate, was assessed through two commonly used bioassays measuring the ability: i) to mobilize iron and ii) to solubilize inorganic phosphorus^28,38,59^. The ability to mobilize iron was tested through the detection of siderophore production on the chrome azurol S (CAS) medium, following the protocol of Frey-Klett *et al*.^67^. This medium is composed of one litre of 800 mL of Solution 1 (34.36 g of Pipe Na_2_ Buffer (Sigma), 0.3 g KH_2_PO_4_, 0.5 g NaCl, 1 g NH_4_Cl, 0.246 g MgSO_4_∙7H_2_O, 0.0147 g CaCl_2_∙2H_2_O and 15 g agar in 800 mL H_2_O; pH 6.8), 100 mL of Solution 2 (4 g glucose in 100 mL H_2_O) and 100 mL of Solution 3 (Mix of 0.0905 g chrome azurol S in 75 mL, 0.0024 g FeCl_3_ in 15 mL, 0.1640 g hexadecyltrimethylammonium bromide in 60 mL H_2_O). Solutions 2 and 3 were added after autoclaving at a temperature of ca. 70 °C. The ability to solubilize inorganic phosphorous was tested using a 1/10 diluted tri-calcium phosphate (TCP) bioassay, according to Lepleux *et al*.^28^. This medium is composed of one litre of distilled water of 0.5 g NH_4_Cl, 0.1 g NaCl, 0.1 g MgSO_4_∙7H_2_O, 1 g glucose, 0.4 g Ca_3_(PO_4_)_2_ and 15 g agar (pH 6.5).

For each bioassay, bacterial isolates were grown for 72 h in 10 mL of liquid lysogeny broth (LB) medium at 25 °C. Two millilitres of each bacterial culture were then collected, washed three times in sterile water and suspended in 2 mL sterile water to obtain a calibrated inoculum suspension at λ 595 nm = 0.50 (ca. 10^7^ cell mL^−1^). After vortexing, 10 µL of this suspension was then used in triplicate for the CAS and TCP assays. After incubation at 25 °C for 7 days, the diameters of colonies and the diameter of the discoloration zone were measured to determine the ability of each bacterial isolate to mobilize iron (CAS) and to solubilize inorganic phosphorous (TCP). The diameter of the discoloration zones was used to determine the relative efficacy of each bacterial isolate in each bioassay and their distribution in the three classes of efficacy based on the size of the discoloration zone on the different media (no mobilization activity, low activity and strong activity). For each medium, the determination of the class of efficacy was performed depending on the median of the discoloration zones of the positive strains (TCP = 1.4 cm; CAS = 1.8 cm) as follows: positive bacterial isolates with discoloration zones lower than the median were classified as bacterial isolates of low activity, whereas bacterial isolates with discoloration zones higher than the median were classified as bacterial isolates of strong activity. A total of 412 and 357 bacterial strains grew on TCP and CAS media, respectively.

### Molecular identification of bacterial isolates

The 16S rRNA gene amplification of the bacterial isolates was performed using the universal set of primers pA [(5′-AGAGTTTGATCCTGGCTCAG-3′) and 907r (5′-CCGTCAATTCMTTTGAGTTT- 3′)^68,69^]. Polymerase chain reactions were performed in a total reaction volume of 50 µL containing 20 µL PCR Master mix (5-PRIME), 2 µL primers (10 µM) and 4 µL of inoculum suspension. The same inoculum suspension used for the functional assays described above was used for molecular characterization. The following temperature cycle was used: initial denaturation for 4 min at 94 °C, followed by 30 cycles of 30 s denaturation at 94 °C, 1 min annealing at 53 °C and 1 min 30 s extension at 72 °C, and a final extension for 10 min at 72 °C. PCR controls with no template or extraction control as a template were negative. PCR products were purified using the QIAquick purification kit (Qiagen, Valencia, CA) as recommended by the manufacturer. The sequencing was performed by MWG Eurofin Operon using the primers described above. The partial 16S rRNA gene sequences generated were compared with those of the GenBank databases using the BLAST programme^70^. They were aligned with reference 16S rRNA gene sequences using SeaView (version 4.5.4^71^). Bootstrap values were generated using 1,000 replicates. Trees were generated using Dendroscope 3 software (version 3.4.4^72^).

### DNA extraction and quantitative PCR

For each treatment (K, Mg fertilized and control) and sampling time, total DNA was extracted from 250 mg of soil. Extractions were performed in three replicates using the ‘PowerSoilTM DNA Isolation Kit’ (MoBio Laboratories, Carlsbad, CA, USA) as recommended by the manufacturer. Concentrations of DNA were measured using a Nanodrop-1000 spectrometer (NanoDrop Technologies, Wilmington, DE, USA). The same quantity of total DNA was used to quantify the total bacterial 16S as well as the bacterial genera (*Burkholderia, Collimonas* and *Pseudomonas*) using the 16S rRNA gene-specific primers [10 μM each; 968 F/1401 R (total bacteria^73^), Burk3/BurkR (*Burkholderia*^74^), Eddy3For/Eddy3Rev/Sophie (*Collimonas*^58^) and PSf/Psr (*Pseudomonas*^75^), respectively]. For T0, T1 and T3, absolute quantifications were performed using serial dilutions of standard plasmids containing total or genus-specific bacterial 16S rDNA inserts (from 10^9^ to 10^2^ gene copies/μl) and the SsoAdvanced Universal SYBR Probes Supermix (classical qPCR for quantification of total bacteria, *Burkholderia* and *Pseudomonas*) and Probes Supermix (TaqMan qPCR for quantification of *Collimonas*) from Bio-Rad. PCR reaction were performed in a final volume of 20 µl containing 10 µl of the mix (SsoAdvanced Universal SYBR Probes Supermix or Probes Supermix), 1 µl of the total DNA, 2 µl of the primers (and of the Taqman probe when necessary) and milliQ water. The different bacterial genera and total bacterial quantifications were performed using the following cycle parameters: 1 cycle of 98 °C for 3 min followed by 40 cycles of 98 °C for 15 s, AT (annealing temperature) °C for 30 s (AT: 56 °C for total bacterial; 64 °C for *Burkholderia*; 63 °C for *Pseudomonas*; 66 °C for *Collimonas*). For each qPCR run using SYBR technology, a melting curve was performed at the end. The population sizes of total bacteria, *Burkholderia*, *Collimonas* and *Pseudomonas*, are expressed as log[number of 16S rRNA gene copies per gram dry weight soil].

### PCR and pyrosequencing analyses

The 16S rRNA gene amplicon libraries were generated in one step using the primers 799 f and 1115r^76,77^ containing the specific Roche 454-pyrosequencing adaptors and 5 bases barcodes, according to Colin *et al*.^34^. PCRs contained 1X PCR Master mix (5 PRIME®), 500 nM 799 f primer, 500 nM 1115r primer and 8 ng of DNA in a final volume of 50 µL. Amplifications were performed using the following cycle parameters: 95 °C for 5 min (initial denaturation), followed by 30 cycles of 95 °C for 30 s, 57 °C for 35 s, and 72 °C for 30 s with a final extension step at 72 °C for 10 min. Triplicate PCR products were generated for each replicate and were then checked by gel electrophoresis, pooled and purified using the QIAquick purification kit (Qiagen, Valencia, CA, USA) as recommended by the manufacturer. The concentration of each purified PCR product was then measured using a Nanodrop-1000 spectrometer (NanoDrop Technologies, Wilmington, DE, USA), and an equimolar mix of the 16S rRNA gene amplicons was used for pyrosequencing on a 454 GS Junior system (Roche; Ecogenomic platform). All the treatments were sequenced, except for the Mg amended condition at T2 and T4. A total of 42,425 raw reads were generated. These 16S rRNA reads were then filtered for length (>300 bp), quality score (mean, ≥25), number of ambiguous bases (=0), and length of homopolymer runs (<8) using the trim.seqs script in Mothur v.1.30.2. These treatments generated a total of 31,216 quality sequences^78^. Chimeric sequences were detected using the chimera.uchime command and were removed from further analysis. To avoid any biases associated with different numbers of sequences in each of the samples, a random subsample of 448 sequences (corresponding to the smaller set of sequences after Mothur processing) from each sample was used for all downstream analyses, leading to a total of 14,784 sequences. High-quality sequences were aligned and clustered into 873 operational taxonomic units (OTUs), defined with 3% dissimilarity level and including 373 singletons. Taxonomy was assigned to each OTU by aligning sequences against the SILVA alignment database (version 128) with a bootstrap value of 80 for taxonomic assignment. The 16S rRNA gene sequences assigned as ‘unclassified bacteria’ with Silva were also verified using the BLAST programme^70^ against the NCBI database. Based on the OTU assignment, library richness and diversity estimates (Chao1, Shannon and inverse Simpson) were calculated in Mothur. In addition, a comparison of the 16S rRNA pyrosequences and Sanger sequences (generated from the culturable representative strains) was performed using the BLAST programme^70^. Alignment was performed with NCBI-BLAST + an e-value cut-off of 1 e-5 and a 100% overlap on the common portion (120 bases). Similar analyses have been performed with the 16S rRNA pyrosequences generated in a previous study on the same soil^40^.

### Statistical analyses

All statistical analyses were performed using R 3.2.3 software^79^. The effect of the treatment and sampling time on the Biolog-substrate utilization profiles and on the respiration rates was determined by analysis of variance (one-factor ANOVA, P < 0.05, followed by a Tukey test). Similarly, ANOVA was conducted to determine the relative efficacy of the bacterial isolates to weather minerals (P < 0.05, followed by a Tukey test). Finally, the hypothesis of an equal distribution of the bacterial isolates according to their efficacy class in the functional assays was tested by a Chi^2^ test (P < 0.05) using the *NCStats* package^80^. Differences in the mineral weathering efficacy of the bacterial strains affiliated with a specific genus were assessed following the same procedure, but only for bacterial genera represented by more than 10 bacterial isolates. Estimation of the taxonomic diversity and richness based on culturable-independent data was carried out with Mothur, followed by statistical analysis (ANOVA followed by a Tukey test, P < 0.05). Permutational multivariate analysis of variance (PERMANOVA) based on Bray-Curtis distances with 999 permutations were performed on the 16S rRNA pyrosequence data using the vegan package^81^. To assess structural differences between bacterial communities of treatments and/or sampling times, relative abundance values at all taxonomic levels (phylum, class, order, family, genus, OTU) were compared for taxa or OTUs presenting a minimum of 100 sequences. These values were transformed using the arcsine square root to achieve a normal distribution. A one-factor ANOVA was then conducted on those data, followed by a Tukey test with a threshold level of P < 0.05. A p adjustment was done using the false-Discovery-Procedure of Benjamini and Hochberg^82^ to determine the p value threshold.

### Nucleotide sequence accession numbers

The sequences determined in this study have been deposited in the GenBank database for the Sanger sequences (MH918162-MH918556) and in the SRA trace archive for the pyrosequences (SRP013944; SRR7812734- SRR7812719).

## Supplementary information

supplementary tables and figures
