# Supplementary material for: A microcosm approach highlights the response of soil mineral weathering bacterial communities to an increase of K and Mg availability
Source: Sci Rep. 2019 Oct 7;9:14403. doi: 10.1038/s41598-019-50730-y (PMC6779897; doi:10.1038/s41598-019-50730-y)
Supplement: Supplementary file 1 — supplementary tables and figures [file 41598_2019_50730_MOESM1_ESM.pdf]

Supplementary files

A microcosm approach highlights the response of soil mineral weathering bacterial communities to an increase of K and Mg availability

AUTHORS: Nicolitch, O.[1,2], Feucherolles, M.[1], Churin, J-L.[1], Fauchery, L.[1], Turpault, M-P.[2], and Uroz, S. [1,2*]

[1] INRA, Université de Lorraine, UMR 1136 "Interactions Arbres Microorganismes", Centre INRA de Nancy, 54280 Champenoux, France

[2] INRA UR 1138 "Biogéochimie des Ecosystèmes Forestiers", Centre INRA de Nancy, 54280 Champenoux, France.

Running title: Shift in bacterial function depending on nutrient availability

* **Corresponding author:** Mailing address:

INRA-Université de Lorraine, Interactions Arbres Microorganismes, UMR 1136, 54280 Champenoux, France. Phone: +33 (0)3 83 39 40 81, Fax: +33 (0)3 83 39 40 69. E-mail: stephane.uroz@inra.fr.

Figure S1 : Respiration measures (expressed as CO2 rate) obtained using the Microresp method on the different treatments (control, K and Mg fertilized) and the four sampling times (T0, T1,T3 and T4).

Microresp data.

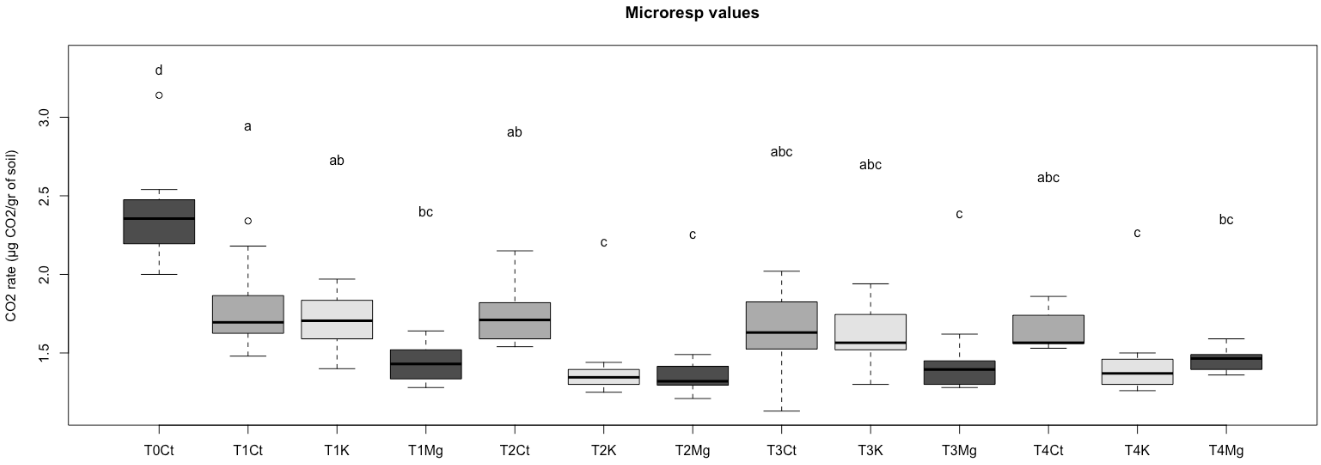

Figure S2 : Relative distribution of the major bacterial phyla in the different treatments (control, K and Mg fertilized) and the four sampling times (T0, T1,T3 and T4).

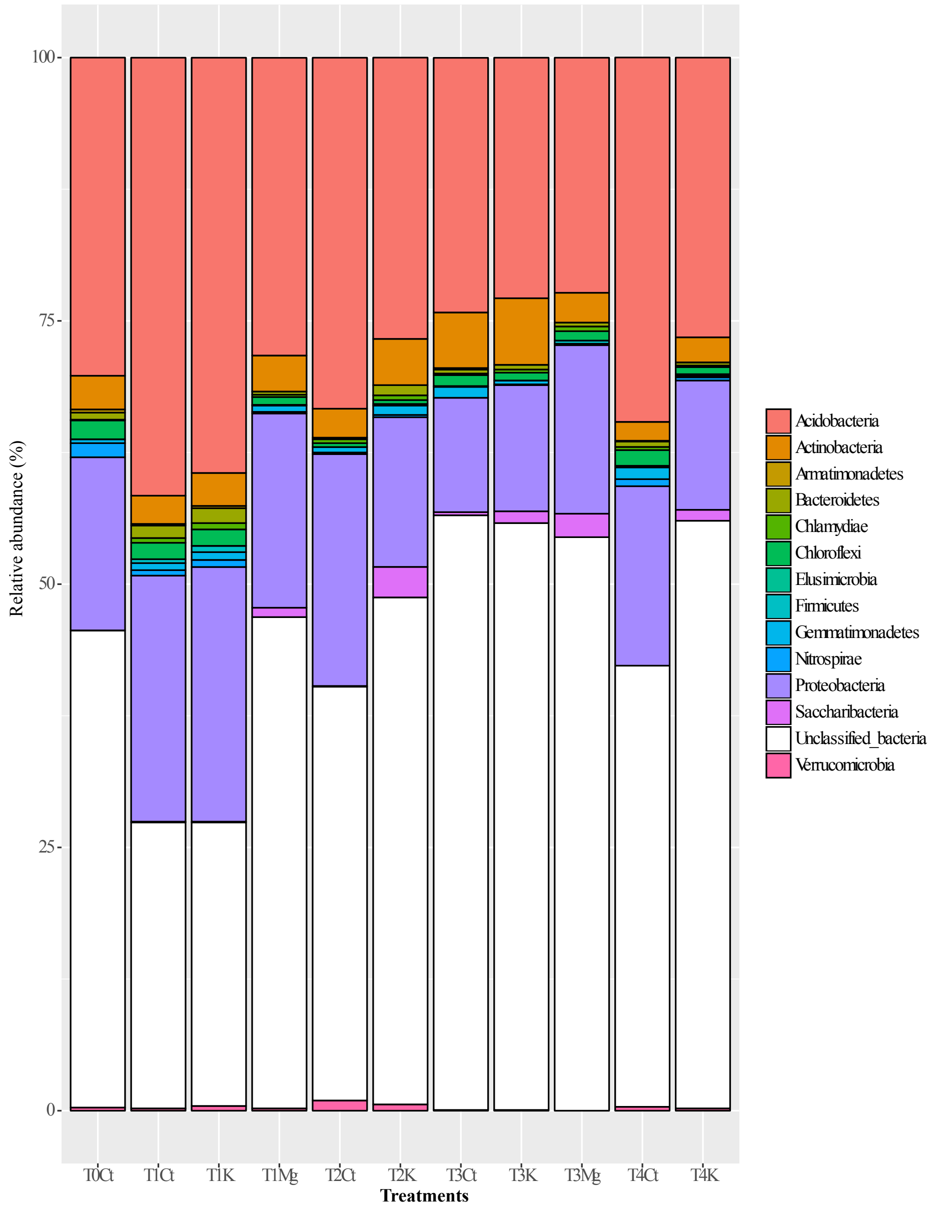

Table S1 : Catabolic potentials measured using the Biolog Ecoplates. For each sampling time (T1 to T4), treatments presenting different letters (a,b,c) are significantly different according to a one-factor ANOVA and Tukey post-hoc test ($P < 0.05$). Treatments are presented as follows: Ct, control; Mg, Mg fertilized and K, K fertilized. The P values presenting asterisk(s) were considered as significant after a FDR correction.

**Substrate categories**	**T1**				**T2**			
Polymers	0.40	NS	Ct = K = Mg	a a a	**0.003**	**	**Ct = K > Mg**	**a a b**
Miscellaneous	0.86	NS	K = Ct = Mg	a a a	0.2	NS	Mg = Ct = K	a a a
Carbohydrates	0.84	NS	Ct = K = Mg	a a a	0.10	NS	Mg = Ct = K	a a a
Carboxylic_acids	0.28	NS	Ct = K = Mg	a a a	**0.009**	**	**Ct = K > Mg**	**a a b**
Amino_acids	0.39	NS	Ct = K = Mg	a a a	0.75	NS	Mg = Ct = K	a a a
**All substrates**	0.64	NS	Ct = K = Mg	a a a	0.17	NS	Mg = Ct = K	a a a

**Substrate categories**	**T3**				**T4**			
Polymers	**0.004**	**	**Mg≥K≥Ct**	**a ab b**	0.025	NS	Mg = K = Ct	a a a
Miscellaneous	0.032	NS	Mg = Ct = K	a a a	0.033	NS	Mg = K = Ct	a a a
Carbohydrates	0.58	NS	Ct = Mg = K	a a a	0.34	NS	Mg = Ct = K	a a a
Carboxylic_acids	0.024	NS	Ct = K = Mg	a a a	0.032	NS	Ct = Mg = K	a a a
Amino_acids	0.5	NS	Ct = Mg = K	a a a	0.81	NS	Mg = Ct = K	a a a
**All substrates**	**0.73**	**N**	Ct = Mg = K	a a a	0.61	NS	Mg = Ct = K	a a a

Table S2: Estimates of the taxonomic diversity in bacterial communities. For each sampling time (T1 to T4), treatments presenting different letters (a,b,c) are significantly different according to a one-factor ANOVA and Tukey post-hoc test (P < 0.05). Treatments are presented as follows: Ct, control; Mg, Mg fertilized and K, K fertilized. Each time treatment is presented (T1 to T4) independently or as factor for comparison. Significant differences are presented in bold. Stars indicate significant differences (***: p < 0.001, **: p < 0.01, *: p < 0.05).

Time	Treatment	Coverage	Sobs	Simpson	Shannon	Chao
**T0**	**Ct**	0.86 ± 0.02 ab	114.67 ± 7.22 a	26.25 ± 4.54 ab	3.89 ± 0.12 a	227.58 ± 31.81 ab
**T1**	**Ct**	0.84 ± 0.01 a	131 ± 3.51 a	45.92 ± 3.18 a	4.23 ± 0.02 a	269.04 ± 15.04 a
	**K**	0.87 ± 0.01 ab	121.33 ± 7.26 a	36.34 ± 1.53 ab	4.13 ± 0.07 a	216.72 ± 15.06 ab
	**Mg**	0.88 ± 0 ab	104 ± 11.93 a	28.15 ± 8.61 ab	3.75 ± 0.32 a	193.44 ± 9.72 ab
**T2**	**Ct**	0.88 ± 0.01 ab	108 ± 5.13 a	28 ± 5.39 ab	3.89 ± 0.13 a	183.43 ± 16.18 ab
	**K**	0.89 ± 0 b	105 ± 1.53 a	22.26 ± 3.36 b	3.77 ± 0.1 a	164.2 ± 4.74 b
**T3**	**Ct**	0.89 ± 0 b	101 ± 2.89 a	15.96 ± 2.13 b	3.6 ± 0.08 a	168.26 ± 9.43 b
	**K**	0.87 ± 0.01 ab	106.33 ± 4.33 a	17.44 ± 5.44 b	3.61 ± 0.18 a	201.81 ± 7.71 ab
	**Mg**	0.89 ± 0.01 b	100.33 ± 8.19 a	16.59 ± 3.02 b	3.59 ± 0.16 a	159.9 ± 19.92 b
**T4**	**Ct**	0.87 ± 0.01 ab	117.67 ± 3.33 a	30.04 ± 1.87 ab	4.02 ± 0.05 a	191.72 ± 20.84 ab
	**K**	0.89 ± 0.02 b	99 ± 12.12 a	19.41 ± 5.21 b	3.59 ± 0.28 a	172.93 ± 28.81 b
**p-value**		**0.028 ***	0.073	**0.002 ****	0.09817	**0.011 ***
**T0**		0.86 ± 0.02 a	114.67 ± 7.22 a	26.25 ± 4.54 ab	3.89 ± 0.12 ab	227.58 ± 31.81 ab
**T1**		0.86 ± 0.01 a	118.78 ± 5.73 a	36.80 ± 3.72 a	4.04 ± 0.12 a	226.40 ± 13.06 a
**T2**		0.88 ± 0.00 a	106.50 ± 2.49 a	25.13 ± 3.12 ab	3.83 ± 0.08 ab	173.82 ± 8.68 ab
**T3**		0.88 ± 0.00 a	102.56 ± 2.96 a	16.66 ± 1.91 b	3.60 ± 0.07 b	176.66 ± 9.30 b
**T4**		0.88 ± 0.01 a	108.33 ± 7.00 a	24.73 ± 3.43 ab	3.81 ± 0.16 ab	182.32 ± 16.45 ab
**p-value**		0.089	0.139	**0.0009 *****	0.06557	**0.014 ***
	**Ct**	0.87 ± 0.01 a	114.47 ± 3.22 a	29.23 ± 2.93 a	3.93 ± 0.06 a	208.01 ± 12.31 a
	**K**	0.88 ± 0.01 a	107.92 ± 4.02 a	23.86 ± 2.86 a	3.78 ± 0.1 a	188.92 ± 9.63 a
	**Mg**	0.89 ± 0.01 a	102.17 ± 6.52 a	22.37 ± 4.83 a	3.67 ± 0.17 a	176.67 ± 12.43 a

p-value	0.134	0.163	0.311	0.210	0.231

Table S3. Comparison of the different treatments at each sampling times at the phylum, class, order, family, genus and OTU levels. The relative distribution of the sequences in the different taxonomic levels considered was analyzed by one-factor ANOVA (and a post-hoc test, P < 0.05) and a P value correction using the False-Discovery-Rate procedure. Only the taxa or OTUs represented by a minimum of 100 sequences have been considered. The symbols '>' mean significantly more abundant and '=' not significantly different. Bold indicates significant difference between the different treatments (Ct, control; K, K amended and Mg, Mg amended). The grey color indicates the sampling times for which the three treatments (Ct, +K, +Mg) have been treated. The number of sequences per taxa considered is presented (N seq). The column 'Level' refers to the taxonomic classification provided by Mothur. The taxonomic affiliation of the different OTUs is presented below the table.

Level	Group	N seq	T0	T1						T2				
				Ct	K	Mg	p-value		Stats	Ct	K	p-value		Stats
2	Saccharibacteria	109	0.07 ± 0.07	0 ± 0 a	0 ± 0 a	0.74 ± 0.41 b	**0.009**	**	**Mg > Ct = K**	0 ± 0	2.83 ± 1.83	**0.048**	**NS**	**K = Ct**
3	Betaproteobacteria	394	2.68 ± 0.34	5.28 ± 0.64	2.9 ± 0.78	1.86 ± 0.91	0.092	NS	Ct = K = Mg	4.46 ± 0.34 b	1.34 ± 0.39 a	**0.007**	**	**Ct > K**
4	Burkholderiales	301	2.23 ± 0.56	4.84 ± 0.41	2.38 ± 0.73	1.04 ± 0.41	**0.014**	**NS**	**Ct = K = Mg**	3.57 ± 0.22 b	0.67 ± 0.34 a	**0.006**	**	**Ct > K**
6	Luteibacter	27	0 ± 0	0.3 ± 0.07	0.22 ± 0.13	0.52 ± 0.52	0.912	NS	Mg = Ct = K	0 ± 0	0 ± 0	1.000	NS	K = Ct
7	Otu0001	3035	12.72 ± 3	6.55 ± 0.88 b	6.55 ± 1.03 a	11.31 ± 4.92 b	**0.005**	***	**K > Ct = Mg**	11.68 ± 2.84	12.28 ± 1.18	0.374	NA	Ct = K
7	Otu0002	1309	7.29 ± 0.71	5.28 ± 1.04 b	7.37 ± 0.46 a	8.78 ± 2.02 a	**0.01**	**	**Ct > K = Mg**	6.18 ± 2.31	12.8 ± 2.39	0.374	NA	Ct = K

Level	Group	N seq	T3						T4				
			Ct	K	Mg	p-value		Stats	Ct	K	p-value		Stats
2	Saccharibacteria	109	0.3 ± 0.3	1.12 ± 0.56	2.31 ± 1.22	0.198	NS	Mg = K = Ct	0.07 ± 0.07	0.67 ± 0.34	0.078	NS	K = Ct
3	Betaproteobacteria	394	1.86 ± 0.97	1.56 ± 0.26	2.01 ± 1.01	0.967	NS	Mg = Ct = K	3.5 ± 0.54	1.86 ± 0.71	0.152	NS	Ct = K
4	Burkholderiales	301	1.41 ± 0.84	1.04 ± 0.2	1.41 ± 0.97	0.982	NS	Ct = Mg = K	2.75 ± 0.37	1.04 ± 0.45	0.067	NS	Ct = K
6	Luteibacter	27	0 ± 0 a	0 ± 0 a	0.74 ± 0.27 b	**0.002**	**	**Mg > Ct = K**	0 ± 0	0.22 ± 0.22	0.374	NS	K = Ct
7	Otu0001	3035	20.68 ± 2.19	20.61 ± 3.74	18.68 ± 4.08	1.000	NS	Mg = K = Ct	11.9 ± 1.07	18.6 ± 3.65	0.374	NA	K = Ct
7	Otu0002	1309	7.81 ± 1.1	8.04 ± 1.56	11.31 ± 1.07	0.083	NS	Ct = K = Mg	6.25 ± 0.68	7.89 ± 3.42	0.374	NA	Ct = K

Taxonomic affiliation of the OTUs

OUT id	Phylum	Class	Order	Family	Genus
Otu0001	Unclassified bacteria	-	-	-	-
Otu0002	Unclassified bacteria	-	-	-	-
Otu0003	Acidobacteria	Subgroup_2	Subgroup_2_or	Subgroup_2_fa	Subgroup_2_ge
Otu0004	Unclassified bacteria	-	-	-	-
Otu0005	Acidobacteria	Subgroup_2	Subgroup_2_or	Subgroup_2_fa	Subgroup_2_ge
Otu0006	Unclassified bacteria	-	-	-	-
Otu0007	Acidobacteria	Acidobacteria	Acidobacteriales	Acidobacteriaceae_(Subgroup_1)	Acidobacteriaceae_(Subgroup_1)_unclassified
Otu0035	Acidobacteria	Acidobacteria	Acidobacteriales	Acidobacteriaceae_(Subgroup_1)	Acidobacteriaceae_(Subgroup_1)_unclassified
Otu0054	Acidobacteria	Acidobacteria_unclassified	Acidobacteria_unclassified	Acidobacteria_unclassified	Acidobacteria_unclassified
Otu0068	Proteobacteria	Alphaproteobacteria	Rhodospirillales	Rhodospirillales_unclassified	Rhodospirillales_unclassified

Table S4: Comparison of the 16S rRNA sequences from the bacterial collection with the amplicon pyrosequencing dataset. For each OTU the percentage of identity with culturable representative of our study is presented as well as the taxonomic affiliation. OTU presenting a strong homology with bacetrial strains are presented in bold.

OTU	Number of sequences	Identity (%)	Phylum	Class	Order	Family	Genus
Otu0001	2037	< 90	Bacteria_unclassified	Bacteria_unclassified	Bacteria_unclassified	Bacteria_unclassified	Bacteria_unclassified
Otu0002	1196	< 90	Bacteria_unclassified	Bacteria_unclassified	Bacteria_unclassified	Bacteria_unclassified	Bacteria_unclassified
Otu0003	781	< 90	Acidobacteria	Subgroup_2	Subgroup_2_or	Subgroup_2_fa	Subgroup_2_ge
Otu0004	779	< 90	Bacteria_unclassified	Bacteria_unclassified	Bacteria_unclassified	Bacteria_unclassified	Bacteria_unclassified
Otu0005	649	< 90	Bacteria_unclassified	Bacteria_unclassified	Bacteria_unclassified	Bacteria_unclassified	Bacteria_unclassified
Otu0006	387	< 90	Acidobacteria	Subgroup_2	Subgroup_2_or	Subgroup_2_fa	Subgroup_2_ge
Otu0007	313	< 90	Acidobacteria	Subgroup_2	Subgroup_2_or	Subgroup_2_fa	Subgroup_2_ge
Otu0008	307	< 90	Acidobacteria	Subgroup_2	Subgroup_2_or	Subgroup_2_fa	Subgroup_2_ge
Otu0009	281	< 90	Bacteria_unclassified	Bacteria_unclassified	Bacteria_unclassified	Bacteria_unclassified	Bacteria_unclassified
Otu0010	280	< 90	Acidobacteria	Acidobacteria	Acidobacteriales	Acidobacteriaceae_(Subgroup_1)	Acidobacteriaceae_(Subgroup_1)_unclassified
Otu0013	186	95.4	Actinobacteria	**Actinobacteria**	**Frankiales**	**Acidothermaceae**	Acidothermus
**Otu0014**	**170**	**99.1**	**Proteobacteria**	**Betaproteobacteria**	**Burkholderiales**	**Burkholderiaceae**	**Burkholderia-Paraburkholderia**
**Otu0019**	**158**	**100**	**Proteobacteria**	**Gammaproteobacteria**	**Xanthomonadales**	**Xanthomonadaceae**	**Xanthomonadaceae_unclassified**
Otu0049	40	92.3	Proteobacteria	**Alphaproteobacteria**	**Rhizobiales**	**Rhizobiales_Incertae_Sedis**	Rhizomicrobium
**Otu0055**	**37**	**100**	**Proteobacteria**	**Betaproteobacteria**	**Burkholderiales**	**Oxalobacteraceae**	**Oxalobacteraceae_unclassified**
Otu0059	35	90.7	Proteobacteria	**Gammaproteobacteria**	**Xanthomonadales**	**Xanthomonadaceae**	Xanthomonadaceae_unclassified
Otu0064	32	89.5	Proteobacteria	**Alphaproteobacteria**	**Rhizobiales**	**Xanthobacteraceae**	Variibacter
Otu0081	22	89.6	Proteobacteria	**Alphaproteobacteria**	**Rhizobiales**	**Rhizobiales_unclassified**	Rhizobiales_unclassified
Otu0089	20	93.6	Actinobacteria	**Actinobacteria**	**Streptomycetales**	**Streptomycetaceae**	Streptacidiphilus
**Otu0090**	**20**	**99**	**Proteobacteria**	**Betaproteobacteria**	**Burkholderiales**	**Burkholderiaceae**	**Burkholderia-Paraburkholderia**
Otu0096	18	92.5	Proteobacteria	**Betaproteobacteria**	**Betaproteobacteria_unclassified**	**Betaproteobacteria_unclassified**	**Betaproteobacteria_unclassified**
**Otu0101**	**17**	**100**	**Actinobacteria**	**Actinobacteria**	**Micrococcales**	**Micrococcaceae**	**Micrococcaceae_unclassified**
Otu0105	17	93.8	Actinobacteria	**Acidimicrobiia**	**Acidimicrobiales**	**uncultured**	**uncultured_ge**
**Otu0108**	**16**	**99.1**	**Proteobacteria**	**Betaproteobacteria**	**Burkholderiales**	**Burkholderiaceae**	**Cupriavidus**
Otu0110	16	94.6	Proteobacteria	**Betaproteobacteria**	**Burkholderiales**	**Comamonadaceae**	**Comamonadaceae_unclassified**
Otu0113	16	89.5	Bacteroidetes	**Sphingobacteriia**	**Sphingobacteriales**	**Chitinophagaceae**	**Chitinophagaceae_unclassified**
Otu0115	15	92.5	Proteobacteria	**Betaproteobacteria**	**Betaproteobacteria_unclassified**	**Betaproteobacteria_unclassified**	**Betaproteobacteria_unclassified**
Otu0117	15	92.7	Proteobacteria	**Gammaproteobacteria**	**Xanthomonadales**	**Xanthomonadaceae**	**Dokdonella**
**Otu0120**	**14**	**100**	**Proteobacteria**	**Betaproteobacteria**	**Burkholderiales**	**Oxalobacteraceae**	**Collimonas**
**Otu0124**	**13**	**99.1**	**Proteobacteria**	**Betaproteobacteria**	**Burkholderiales**	**Oxalobacteraceae**	**Oxalobacteraceae_unclassified**
Otu0127	12	96.2	Proteobacteria	**Betaproteobacteria**	**Nitrosomonadales**	**Nitrosomonadaceae**	**Nitrosospira**
Otu0130	12	91.8	Bacteria_unclassified	**Bacteria_unclassified**	**Bacteria_unclassified**	**Bacteria_unclassified**	**Bacteria_unclassified**
Otu0133	12	89.8	Proteobacteria	**Gammaproteobacteria**	**Xanthomonadales**	**Xanthomonadaceae**	**Xanthomonadaceae_unclassified**

**Otu0144**	**10**	**100**	**Proteobacteria**	**Gammaproteobacteria**	**Xanthomonadales**	**Xanthomonadaceae**	**Xanthomonadaceae_unclassified**
Otu0150	10	91.3	Proteobacteria	**Alphaproteobacteria**	**Rhizobiales**	**Roseiarcaceae**	**Roseiarcus**
Otu0158	9	93.5	Actinobacteria	**Actinobacteria**	**Actinobacteria_unclassified**	**Actinobacteria_unclassified**	**Actinobacteria_unclassified**
**Otu0164**	**9**	**98.1**	**Proteobacteria**	**Betaproteobacteria**	**Burkholderiales**	**Burkholderiaceae**	**Burkholderia-Paraburkholderia**
Otu0165	9	91.3	Proteobacteria	**Alphaproteobacteria**	**Rhizobiales**	**Xanthobacteraceae**	**uncultured**
Otu0167	9	91.7	Actinobacteria	**Actinobacteria**	**Actinobacteria_unclassified**	**Actinobacteria_unclassified**	**Actinobacteria_unclassified**
Otu0168	8	92.3	Bacteroidetes	**Sphingobacteriia**	**Sphingobacteriales**	**Sphingobacteriaceae**	**Mucilaginibacter**
**Otu0192**	**7**	**98.1**	**Proteobacteria**	**Betaproteobacteria**	**Burkholderiales**	**Oxalobacteraceae**	**Oxalobacteraceae_unclassified**
Otu0193	6	91.3	Proteobacteria	Alphaproteobacteria	Rhizobiales	Rhizobiales_unclassified	Rhizobiales_unclassified
Otu0194	6	92.2	Proteobacteria	Alphaproteobacteria	Rhizobiales	Xanthobacteraceae	uncultured
Otu0199	6	91.3	Proteobacteria	Alphaproteobacteria	Alphaproteobacteria_unclassified	Alphaproteobacteria_unclassified	Alphaproteobacteria_unclassified
Otu0208	6	91.4	Proteobacteria	Alphaproteobacteria	Rhizobiales	Bradyrhizobiaceae	Bradyrhizobiaceae_unclassified
**Otu0212**	**6**	**99**	**Proteobacteria**	**Gammaproteobacteria**	**Xanthomonadales**	**Xanthomonadaceae**	**Xanthomonadaceae_unclassified**
Otu0213	6	91.7	Actinobacteria	Actinobacteria	Actinobacteria_unclassified	Actinobacteria_unclassified	Actinobacteria_unclassified
**Otu0226**	**5**	**99**	**Proteobacteria**	**Betaproteobacteria**	**Burkholderiales**	**Burkholderiaceae**	**Burkholderia-Paraburkholderia**
Otu0232	5	92.6	Proteobacteria	Betaproteobacteria	Burkholderiales	Comamonadaceae	Aquincola
Otu0233	5	90.8	Actinobacteria	Actinobacteria	Actinobacteria_unclassified	Actinobacteria_unclassified	Actinobacteria_unclassified
Otu0239	5	95.2	Bacteria_unclassified	Bacteria_unclassified	Bacteria_unclassified	Bacteria_unclassified	Bacteria_unclassified
**Otu0243**	**5**	**99.1**	**Actinobacteria**	**Actinobacteria**	**Streptomycetales**	**Streptomycetaceae**	**Streptomycetaceae_unclassified**
Otu0245	5	91.6	Proteobacteria	Betaproteobacteria	Betaproteobacteria_unclassified	Betaproteobacteria_unclassified	Betaproteobacteria_unclassified
Otu0261	4	89.8	Proteobacteria	Gammaproteobacteria	Xanthomonadales	Xanthomonadaceae	Rhodanobacter
Otu0264	4	91.3	Proteobacteria	Alphaproteobacteria	Rhizobiales	Xanthobacteraceae	uncultured
Otu0269	4	91.9	Actinobacteria	Actinobacteria_unclassified	Actinobacteria_unclassified	Actinobacteria_unclassified	Actinobacteria_unclassified
Otu0272	4	93.2	Proteobacteria	Alphaproteobacteria	Rhizobiales	Rhizobiales_unclassified	Rhizobiales_unclassified
Otu0279	4	91.4	Bacteria_unclassified	Bacteria_unclassified	Bacteria_unclassified	Bacteria_unclassified	Bacteria_unclassified
Otu0282	4	90	Actinobacteria	Acidimicrobiia	Acidimicrobiales	uncultured	uncultured_ge
Otu0285	4	91.7	Proteobacteria	Gammaproteobacteria	Xanthomonadales	Xanthomonadaceae	Xanthomonadaceae_unclassified
Otu0289	4	93.6	Actinobacteria	Actinobacteria	Actinobacteria_unclassified	Actinobacteria_unclassified	Actinobacteria_unclassified
Otu0295	4	92.7	Actinobacteria	Actinobacteria	Corynebacteriales	Nocardiaceae	Nocardia
Otu0299	4	92.6	Actinobacteria	Actinobacteria	Frankiales	Frankiales_unclassified	Frankiales_unclassified
Otu0302	4	93.3	Proteobacteria	Alphaproteobacteria	Rhizobiales	Rhizobiales_unclassified	Rhizobiales_unclassified
Otu0316	3	93.5	Firmicutes	Bacilli	Bacillales	Paenibacillaceae	Cohnella
**Otu0320**	**3**	**99.1**	**Firmicutes**	**Bacilli**	**Bacillales**	**Bacillaceae**	**Bacillus**
**Otu0324**	**3**	**97.4**	**Bacteria_unclassified**	**Bacteria_unclassified**	**Bacteria_unclassified**	**Bacteria_unclassified**	**Bacteria_unclassified**
Otu0335	3	95.4	Firmicutes	Bacilli	Lactobacillales	Lactobacillaceae	Lactobacillus
Otu0340	3	89.8	Actinobacteria	Acidimicrobiia	Acidimicrobiales	uncultured	uncultured_ge
Otu0357	3	91.1	Actinobacteria	Acidimicrobiia	Acidimicrobiales	uncultured	uncultured_ge
Otu0367	3	93.8	Actinobacteria	Acidimicrobiia	Acidimicrobiales	uncultured	uncultured_ge
Otu0385	2	91.6	Proteobacteria	Betaproteobacteria	Betaproteobacteria_unclassified	Betaproteobacteria_unclassified	Betaproteobacteria_unclassified
**Otu0389**	**2**	**98.1**	**Firmicutes**	**Bacilli**	**Bacillales**	**Paenibacillaceae**	**Paenibacillus**
Otu0395	2	95.4	Actinobacteria	Actinobacteria	Micrococcales	Microbacteriaceae	Microbacteriaceae_unclassified

Otu0408	2	93.1	Proteobacteria	Alphaproteobacteria	Rhizobiales	Phyllobacteriaceae	Phyllobacteriaceae_unclassified
**Otu0414**	**2**	**98.1**	**Bacteroidetes**	**Sphingobacteriia**	**Sphingobacteriales**	**Sphingobacteriaceae**	**Pedobacter**
**Otu0420**	**2**	**98.2**	**Actinobacteria**	**Actinobacteria**	**Streptomycetales**	**Streptomycetaceae**	**Streptomycetaceae_unclassified**
Otu0426	2	95.3	Firmicutes	Bacilli	Bacillales	Paenibacillaceae	Paenibacillaceae_unclassified
Otu0430	2	90.2	Proteobacteria	Alphaproteobacteria	Rhizobiales	Hyphomicrobiaceae	Hyphomicrobiaceae_unclassified
Otu0438	2	91.3	Proteobacteria	Alphaproteobacteria	Rhizobiales	Rhizobiales_unclassified	Rhizobiales_unclassified
**Otu0449**	**2**	**97.2**	**Firmicutes**	**Bacilli**	**Bacillales**	**Planococcaceae**	**Planococcaceae_unclassified**
**Otu0453**	**2**	**99.1**	**Bacteria_unclassified**	**Bacteria_unclassified**	**Bacteria_unclassified**	**Bacteria_unclassified**	**Bacteria_unclassified**
Otu0491	2	91.1	Actinobacteria	Actinobacteria_unclassified	Actinobacteria_unclassified	Actinobacteria_unclassified	Actinobacteria_unclassified
**Otu0493**	**2**	**99.1**	**Proteobacteria**	**Betaproteobacteria**	**Burkholderiales**	**Oxalobacteraceae**	**Oxalobacteraceae_unclassified**
Otu0494	2	91.2	Actinobacteria	Acidimicrobiia	Acidimicrobiales	Acidimicrobiales_unclassified	Acidimicrobiales_unclassified
Otu0517	1	90.4	Bacteroidetes	Sphingobacteriia	Sphingobacteriales	Sphingobacteriaceae	Mucilaginibacter
**Otu0563**	**1**	**98.1**	**Proteobacteria**	**Gammaproteobacteria**	**Pseudomonadales**	**Pseudomonadaceae**	**Pseudomonas**
**Otu0575**	**1**	**99**	**Firmicutes**	**Bacilli**	**Bacillales**	**Paenibacillaceae**	**Paenibacillus**
Otu0584	1	91.8	Bacteria_unclassified	Bacteria_unclassified	Bacteria_unclassified	Bacteria_unclassified	Bacteria_unclassified
**Otu0589**	**1**	**98.1**	**Proteobacteria**	**Betaproteobacteria**	**Burkholderiales**	**Oxalobacteraceae**	**Oxalobacteraceae_unclassified**
Otu0590	1	93.6	Actinobacteria	Actinobacteria	Actinobacteria_unclassified	Actinobacteria_unclassified	Actinobacteria_unclassified
Otu0595	1	95.6	Actinobacteria	Actinobacteria	Micrococcales	Micrococcaceae	Micrococcaceae_unclassified
Otu0609	1	91.4	Proteobacteria	Betaproteobacteria	Betaproteobacteria_unclassified	Betaproteobacteria_unclassified	Betaproteobacteria_unclassified
Otu0614	1	94.4	Actinobacteria	Actinobacteria	Streptosporangiales	Thermomonosporaceae	Thermomonosporaceae_unclassified
**Otu0620**	**1**	**97.4**	**Bacteria_unclassified**	**Bacteria_unclassified**	**Bacteria_unclassified**	**Bacteria_unclassified**	**Bacteria_unclassified**
Otu0644	1	93.8	Actinobacteria	Acidimicrobiia	Acidimicrobiales	uncultured	uncultured_ge
Otu0652	1	91.5	Proteobacteria	Betaproteobacteria	Betaproteobacteria_unclassified	Betaproteobacteria_unclassified	Betaproteobacteria_unclassified
Otu0670	1	95.2	Bacteria_unclassified	Bacteria_unclassified	Bacteria_unclassified	Bacteria_unclassified	Bacteria_unclassified
Otu0681	1	91.7	Actinobacteria	Actinobacteria	Corynebacteriales	Corynebacteriales_unclassified	Corynebacteriales_unclassified
Otu0685	1	92.4	Actinobacteria	Thermoleophilia	Gaiellales	uncultured	uncultured_ge
Otu0712	1	90.4	Proteobacteria	Alphaproteobacteria	Alphaproteobacteria_unclassified	Alphaproteobacteria_unclassified	Alphaproteobacteria_unclassified
Otu0720	1	91.7	Actinobacteria	Actinobacteria	Actinobacteria_unclassified	Actinobacteria_unclassified	Actinobacteria_unclassified
Otu0728	1	92.7	Actinobacteria	Actinobacteria	Frankiales	Acidothermaceae	Acidothermus
Otu0730	1	94.6	Proteobacteria	Betaproteobacteria	Burkholderiales	Burkholderiales_unclassified	Burkholderiales_unclassified
**Otu0751**	**1**	**98**	**Firmicutes**	**Bacilli**	**Bacillales**	**Paenibacillaceae**	**Paenibacillaceae_unclassified**
Otu0759	1	94.4	Firmicutes	Bacilli	Lactobacillales	Lactobacillales_unclassified	Lactobacillales_unclassified
Otu0769	1	92.3	Bacteroidetes	Sphingobacteriia	Sphingobacteriales	Sphingobacteriaceae	Sphingobacteriaceae_unclassified
Otu0772	1	94.6	Proteobacteria	Betaproteobacteria	Burkholderiales	Comamonadaceae	Comamonadaceae_unclassified
**Otu0776**	**1**	**99**	**Firmicutes**	**Bacilli**	**Bacillales**	**Paenibacillaceae**	**Paenibacillus**
Otu0822	1	96.7	Firmicutes	Bacilli	Bacillales	Bacillales_unclassified	Bacillales_unclassified
**Otu0829**	**1**	**99.1**	**Firmicutes**	**Bacilli**	**Bacillales**	**Paenibacillaceae**	**Paenibacillus**
Otu0831	1	92	Actinobacteria	Acidimicrobiia	Acidimicrobiales	Acidimicrobiales_unclassified	Acidimicrobiales_unclassified
**Otu0866**	**1**	**98.1**	**Proteobacteria**	**Alphaproteobacteria**	**Rhizobiales**	**Rhizobiaceae**	**Rhizobium**
